# Local false discovery rate and minimum total error rate approaches to identifying interesting chromosomal regions

**DOI:** 10.1186/1471-2156-6-S1-S23

**Published:** 2005-12-30

**Authors:** Ritwik Sinha, Moumita Sinha, George Mathew, Robert C Elston, Yuqun Luo

**Affiliations:** 1 Department of Epidemiology and Biostatistics, Case Western Reserve University, Cleveland, Ohio; 2 Department of Mathematics, Southwest Missouri State University, Springfield, Missouri

## Abstract

The simultaneous testing of a large number of hypotheses in a genome scan, using individual thresholds for significance, inherently leads to inflated genome-wide false positive rates. There exist various approaches to approximating the correct genomewide *p*-values under various assumptions, either by way of asymptotics or simulations. We explore a philosophically different criterion, recently proposed in the literature, which controls the false discovery rate. The test statistics are assumed to arise from a mixture of distributions under the null and non-null hypotheses. We fit the mixture distribution using both a nonparametric approach and commingling analysis, and then apply the local false discovery rate to select cut-off points for regions to be declared interesting. Another criterion, the minimum total error, is also explored. Both criteria seem to be sensible alternatives to controlling the classical type I and type II error rates.

## Background

The increase in genome-wide experiments and sequencing of multiple genomes has resulted in the analysis of large data sets that involve the simultaneous testing of statistical hypotheses on a large number of features in a genome. Traditionally, researchers have tackled the problem of multiple testing in linkage analysis by proposing a common threshold to control the family-wise (i.e., genome-wide) error rate. The simplest and frequently used Bonferroni correction is often conservative and is mainly useful when the number of tests involved is not very large. Recently, new criteria have been proposed, the false discovery rate [1] being one of them. The large number of hypotheses can be looked upon as coming from a mixture of null and non-null hypotheses. Linkage analysis is then a matter of assigning categories after fitting a mixture to the linkage signals.

A nonparametric empirical Bayes approach that makes simultaneous inferences based on *z*-values (standard normal deviates), converted from the *p*-values of the test statistics has been introduced [2,3]. The authors have explored a philosophically different approach that does not claim whether a test is significant or not, but rather whether a result is "interesting" or "uninteresting". The problem of multiple testing is addressed via the concept of the local false discovery rate (LFDR). Let the prior proportion of "uninteresting" hypotheses (e.g., absence of any major gene effect) be *p*_o_, with corresponding density for the *z*-values being *f*_o_(*z*), and let the "interesting" hypotheses (e.g., presence of a major gene effect) have a prior proportion *p*_1_, with corresponding density for the *z*-values being *f*_1_(*z*). Thus, *f*(*z*) = *p*_o _*f*_o_(*z*) + *p*_1 _*f*_1_(*z*) gives the distribution of the *z*-values under the mixture of the populations of interesting and uninteresting hypotheses. The posterior probability for a particular *z*-value,*z*, to be from an uninteresting hypothesis, is *p*_0_*f*_0_(*z*)/*f*(*z*), and that to be from an interesting hypothesis is 1 - *p*_o _*f*_o_(*z*)/*f*(*z*) The LFDR is defined to be fdr(*z*) = *f*_0_(*z*)/*f*(*z*), which should be very close to the posterior probability P(hypothesis is uninteresting | *z*) = *p*_0 _*f*_0_(*z*)/*f*(*z*) under the often valid assumption that *p*_0 _is close to 1. In Efron [2], all the *z*-values for which fdr(z) ≤ 0.10 are declared to be from interesting hypotheses. A threshold of 0.10 or smaller is desirable in that the proportion of false discoveries will in this way be controlled. We have devised another criterion, the minimum total error (MTE), for identifying "interesting" regions in the genome. This method is explained in the methods section.

Efron [2] estimated the mixture distribution *f*(*z*) nonparametrically by fitting a Poisson regression to the *z*-values. In addition to implementing this approach, we also estimate the mixture distribution parametrically. For the nonparametric approach, a natural spline, as proposed by Efron [2], is fitted to the *z*-values resulting from the genome scan. The normal empirical null is then estimated from the central peak. The results of the analysis can change drastically depending on whether the null distribution is set to be the usual N(0, 1) or is estimated from the data. We use the notation N-LFDR for the procedure of estimating the mixture nonparametrically via a spline and then applying the LFDR to determine the cut-off point.

We also propose, as an alternative, to assume that both *f*_0_(*z*) and *f*_1_(*z*) are normal distributions with common variances but a larger mean for *f*_1_(*z*). This is a common practice in statistics as a first step in comparing two treatments, with the difference in the means of the normals being the treatment effect. After fitting the mixture distribution via commingling analysis as implemented in S.A.G.E. [4], both the LFDR and the MTE criteria were applied to determine the cut-off for claiming a chromosomal region as being interesting. These two approaches are referred to as parametric-local false discover rate (P-LFDR) and parametric-minimum total error (P-MTE), respectively, in what follows.

## Methods

### Data and linkage model

Linkage analysis was performed on the Kofendrerd Personality Disorder (KPD) data with disease being expressed as a binary trait. The true regions causing the disease were known. This study is based on the nuclear family data available from the Aipotu (AI), Karangar (KA), and Danacaa (DA) populations. The analysis was performed on both the microsatellite (MS) and the single-nucleotide polymorphism (SNP) data sets. For the parametric approaches, ten replicates (2, 14, 17, 23, 35, 42, 67, 84, 85, and 90), were randomly chosen and analyzed. Another ten replicates of the AI population (1, 26, 48, 50, 51, 60, 65, 88, 93, and 95) were used in calculations studying the effects of increasing sample size. For the N-LFDR, we used all twenty of the above replicates. All methods of analysis were performed on each population, for the SNPs and the MS markers separately.

In each case, the Haseman-Elston [5] regression model as extended by Shete et al. [6] was fitted using the W4 option of SIBPAL in S.A.G.E. 4.5[4]. In this approach, the dependent variable is a weighted combination of the squared difference and squared mean-corrected sum of the sibling trait values. The *p*-values for a whole multipoint genome scan at 2-cM intervals were converted to standard normal *z*-values, that is, *z *= Φ^-1^(1-*p*), where Φ(●) is the standard normal cumulative distribution function, so that interesting hypotheses are more likely to produce larger *z*-values.

### The N-LFDR approach

For the N-LFDR, the *z*-values were plotted in a histogram of 60 equi-length intervals, each of 0.1 unit. Then a natural spline of degree seven was fitted to the histogram (which has been shown in Efron [2] to be equivalent to fitting a Poisson regression to estimate the expected number of counts in each interval into which the data have been partitioned) to obtain , the estimate of the mixture distribution. A spline of degree seven has the same degrees of freedom as a polynomial of degree six. To fit a bimodal curve a polynomial of degree at least six is needed. Generalized cross-validation [7] was used to evaluate the choice of the degree for fitting a spline. We applied this procedure to both the MS and SNP data of replicate 85. For the MS data, both AI and KA showed the optimal degrees to be approximately seven. The optimal degrees for the other data were higher than seven and were all different. For uniformity we decided to fit a spline of degree seven in every case. SPLUS was used to fit the mixture distribution.

The empirical null distribution, *f*_0_(*z*) is assumed to be normal with mean *δ*_0 _and variance *σ_0_^2^*. These parameters were estimated from the central peak of the observed mixture distribution. Specifically, the mean is estimated by the mode of the spline,  = argmax . A potential estimate for the standard deviation is



However, this estimate is numerically unstable and a smoothing technique, as suggested by Efron [2], was used instead. A quadratic equation of the form  (where *x*_*k *_is the median of the *k*^th ^of 30 0.1-unit intervals within 1.5 units of *δ*_0_) was fitted by least squares, and the estimated standard deviation is . Table 1 shows the parameter estimates of the null distributions for the different populations using SNPs and MSs from replicate 85. The N-LFDR is estimated as  and those chromosomal locations for which the N-LFDR is less than or equal to 0.1 were deemed interesting.

### The P-LFDR and P-MTE approaches

Commingling analysis was performed on the *z*-values using the SEGREG program in S.A.G.E. 4.5 [4]. Initially, a Box-Cox transformation [8] was attempted. However, this produced a density  with a smaller mean than that of . Thus, *z*-values less than – 2, which are classified as uninteresting, were trimmed off to allow the fitting. The percentage of *z*-values below -2 was on average 1%. Promising results were then found after fitting a mixture of two normal distributions without any transformation and with both means estimated from the data. The variances were constrained to be equal to avoid numerical instability.

**Figure 1:**
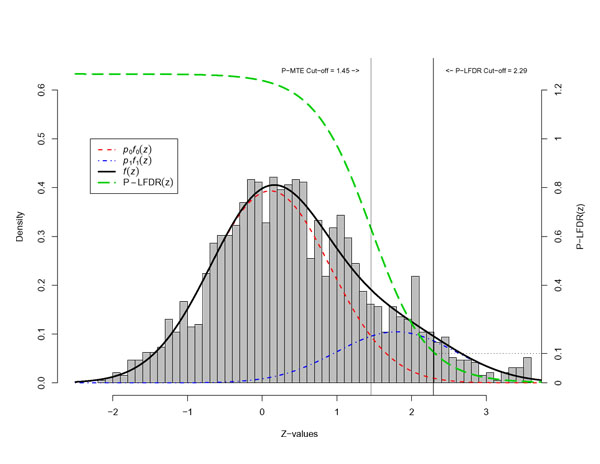
The P-MTE and P-LFDR methods applied to the DA population, replicate 14, MS data.

After fitting the mixture distribution, a criterion is called for to identify z-values that are likely to be classified as interesting, i.e., locations that are potentially genetically linked to disease causing regions. Two criteria, the P-LFDR and the P-MTE, were explored for this purpose. The P-MTE method sets the cut-off at the intersection of the fitted null and alternative distributions. Figure 1 illustrates this method. If x_0 _is the intersection point of  and , then locations with z-values > x_0 _are declared interesting. In Figure 1, x_0 _= 1.45. Note that the area under the null distribution curve to the right of this point is the probability of type I error, that is, we make an error by declaring an underlying uninteresting region as interesting. Similarly, the area under the alternative distribution curve to the left of this point is the probability of type II error, that is, genuine interesting regions are declared uninteresting. The P-MTE method minimizes the total probability of these two types of errors. Note that the P-MTE criterion implicitly assigns equal costs to committing either type of error, which may not be desirable. The P-LFDR criterion, on the other hand, assumes most of the large number of hypotheses are uninteresting and controls the false discovery rate. As for N-LFDR, we declared a region as interesting when fdr(z) < 0.1.

## Results

For each population in each replicate, analysis yields p-values at 1,921 locations on the ten chromosomes with the MS data, and at 2,380 locations with the SNP data. The p-values were converted to z-values by inverting the distribution function of a standard normal. Mixture distributions were fitted to the z-values from each population, each replicate, separately for each type of marker data. Both the nonparametric and the parametric approaches were used in the fitting. The interesting regions obtained were compared with the true regions. The disease causing loci are D1, D2, D3, and D4 for the AI and KA populations and D1 and D2 for the DA population. The modifying loci, D5 and D6, were not considered as disease loci, as our focus is to compare the performance of the new criteria rather than to seek the best linkage analysis model. We defined a location as being "linked" to the disease if it is within 10 cM of a disease locus. We defined loci more than 20 cM from any disease locus as "unlinked". Chromosomal regions between 10 cM and 20 cM away from any disease locus were intentionally ignored to allow for a sharp distinction between "linked" and "unlinked" cases.

**Figure 2:**
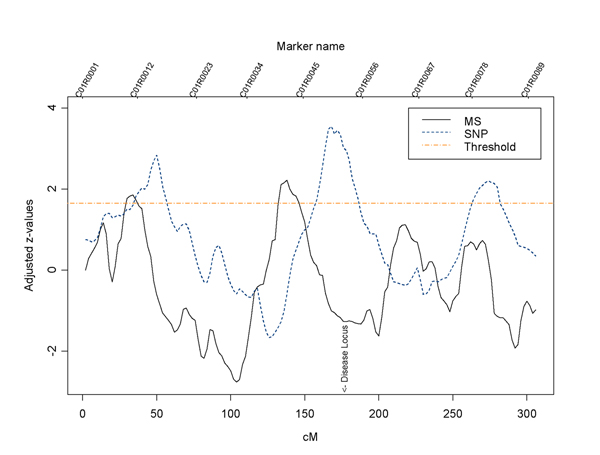
Comparison of linkage signals between data with MS and SNP markers using the N-LFDR (DA population, replicate 85).

For N-LFDR, where the mixture distribution is fitted by a spline, in all the replicates of the DA population the true regions were identified 39.52% of the time using the MSs and 46.67% of the times using the SNPs. Given that the SNPs are more than twice as dense as the MSs, the power of using either is comparable in our data. Figure 2 shows the z-values on chromosome 1 for the MS and SNP data from the DA population in replicate 85. The thresholds for declaring interesting regions are different in the two datasets. Thus we shifted the z-values for the MS data down by 1.1 to equate the thresholds presented in Figure 2. In this replicate the MS marker data were unable to identify D1. The figure shows that MSs and SNPs do not always yield concordant results. Also, it appears that the SNPs yield more accurate localizations than the MSs because the peak of the signal using MS markers is shifted to the left. However, the method was not very successful in identifying the true disease locations for the AI population, possibly due to a relatively more complicated disease model and the lack of covariates in the linkage model.

The parametric approach fitted the mixture distributions to the z-values via commingling analysis. Both the P-MTE and the P-LFDR criteria were applied to the fitted mixture distributions to determine the threshold on the z-values for declaring a genome location interesting. The results for the DA population with MS data are shown in Figure 1. The former gives a threshold of 1.45, while the latter gives one of 2.29.

For each of the three populations and each of the two types of marker data, the probability of committing each of the two types of errors, pooled over all replicates considered, has been calculated and is reported in Table 2. The P-MTE method, as expected, minimizes the total probabilities of the two types of errors, while the P-LFDR more effectively minimizes type I error. For both methods, the SNP data yielded a relatively smaller type II error rate and a larger type I error rate compared with those from the MS data. Overall, the DA population has the smallest error rates, which is not surprising because the genetic effects are better defined and more evident in this population.

We also investigated whether increasing the sample size will provide better results. For this, we merged replicates to form larger samples and repeated the analysis on these samples. The results on the AI population are reported in Tables 3 and 4. Specifically, there are 20 replicates, each of 100 pedigrees. To make samples of 200 pedigrees, pairs of replicates are merged to yield 10 such samples. Five 400-pedigree samples are obtained by merging 4 replicates. Similar analysis was performed each time on the merged datasets.

The results for N-LFDR are provided in Table 3. With increasing sample size, the true regions are discovered with higher probabilities. The method also then controlled the false positive rates considerably. The false positive rate for the data with 400 pedigrees appears to be higher than that for samples with 200 pedigrees (0.29% versus 0.47%). However, not much confidence can be placed on estimates of small probabilities for a binomial distribution in small samples, as the standard deviation is several times the magnitude of the estimate. What is clear is that the type I error rates are quite small for all three sample sizes.

Results for the parametric approach are presented in Table 4. Type I error rates were reduced considerably for both P-MTE and P-LFDR when the sample size increased from 100 to 200, and the improvement is more dramatic when the sample size increased from 200 to 400. Power improves considerably as sample size increases from 100 to 200, but does not see an improvement when the sample size is further increased to 400. This again may be due to the fact that we have only five 400-pedigree samples.

## Discussion

Appropriate control of various types of error rates in multiple testing scenarios has long been an intriguing research problem. However, despite the immense efforts spent on this subject, satisfactory solutions are not available. For example, the application of the genome-wide significance criterion suggested by Lander and Kruglyak [9] to the simulated dataset yielded extremely conservative results.

In this article, we explored the control of false discovery rates, a philosophically different approach, instead of the classical type I and type II error rates in multiple testing problems. Individual test statistics, after appropriate scaling, are viewed as having arisen from a mixture of null and non-null hypotheses. The ratio of the null density versus the mixture density at a given test statistic provides a measure of the LFDR. A procedure that places a cut-off on this ratio controls the FDR.

The mixture distributions were fitted using two different procedures: by fitting 1) a spline (N-LFDR), and 2) a mixture of normal distributions with differing means (P-MTE and P-LFDR). A direct comparison of these approaches with methods in the literature that control the classical types of error rates is not necessarily meaningful because of the philosophical difference between them. Both the local FDR and the MTE criteria have intuitively simple and appealing interpretations.

P-MTE and P-LFDR were compared in terms of finding genuine regions containing disease genes. We observed that the P-LFDR method is more conservative than the P-MTE method in declaring a location "interesting". Therefore, while fewer type I errors are incurred, more truly interesting locations are missed. Table 2 indicates that the SNP data produced greater type I error rates and smaller type II error rates compared with the MS data using the same linkage analysis method. This might be due to that SNPs and MS data have different information content.

The DA population had a simpler genetic model and hence the associated genes were identified with lower type I and type II error rates than in the other populations, as expected. As the sample size increases (Tables 3 and 4) the power increases, which we would expect from any good criterion and analysis method. In N-LFDR we also observe that the type I error remains the same for samples of 100 pedigrees and 200 pedigrees, but there is an increase in the type I error in the samples of 400 pedigrees. This may be due to the fact that there were only five samples of 400 pedigrees. With increasing sample size in the parametric approach both types of error rate decrease, confirming that the two distributions in the mixture separate further with increasing sample size. Note that we are estimating small binomial probabilities, which have standard errors many times the mean and hence these estimates should not be trusted when the sample size is small.

Finally, although both methods for fitting the mixture distributions implicitly assume that the test statistics are independent, which is surely violated in a true multipoint genome scan, the estimate of the mixture distribution is nevertheless consistent and requires a large sample size to be accurate.

## Abbreviations

KPD: Kofendrerd Personality Disorder

LFDR: Local false discovery rate

MS: Microsatellite

MTE: Minimum total error

P-LFDR: Parametric-local false discover rate

P-MTE: Parametric-minimum total error

SNP: Single-nucleotide polymorphism

## Authors' contributions

RS, MS, and GM are equally involved in the statistical analysis, interpretation, preparation of the draft and in the final revision of the manuscript. RCE and YL contributed to the initial conception and design, and critical revision of the manuscript. All authors approved of the manuscript.

## Figures and Tables

**Table 1:** Estimates of means and standard deviations of the empirical null distributions (replicate 85, N-LFDR).

Marker type	Population (SD)		
	
	AI	DA	KA
MS	-0.05 (1.20)	-0.15 (0.95)	0.45 (1.03)
SNP	-0.15 (1.03)	-0.45 (0.89)	0.25 (0.88)

**Table 2:** Estimated probabilities of the two types of errors, with criteria P-MTE and P-LFDR.

Population	Marker	P-MTE (%)		P-LFDR(%)	
			
		Type I error rate	Type II error rate	Type I error rate	Type II error rate
AI	MS	10.94	30.0	3.87	47.5
	SNP	19.43	17.5	6.55	30.0
DA	MS	7.49	25.0	2.85	35.0
	SNP	9.61	0.0	4.80	15.0
KA	MS	12.67	22.5	3.37	57.5
	SNP	13.37	20.0	4.51	37.5

**Table 3:** Efficiency of N-LFDR versus sample size (AI population, MS markers, N-LFDR).

Number of pedigrees per sample	Type I error rate (%)	1- Type II error rate (power) (%)
100	0.29	21
200	0.29	55
400	0.47	90

**Table 4:** Effect of sample size on the error rates (AI population, MS marker, parametric approaches with criteria P-MTE and P-LFDR).

Sample size	P-MTE(%)		P-LFDR (%)	
	
	Type I error rate	1-Type II error rate (power)	Type I error rate	1-Type II error rate (power)
100	10.94	70.0	3.87	52.5
200	9.42	92.5	2.10	77.5
400	1.77	90.0	0.57	70.0
